# The genome sequence of the stilt bug, *Berytinus montivagus* (Meyer-Dur, 1841) (Hemiptera: Berytidae)

**DOI:** 10.12688/wellcomeopenres.28016.1

**Published:** 2026-10-02

**Authors:** Liam M. Crowley, University of Oxford and Wytham Woods Genome Acquisition Lab, Darwin Tree of Life Barcoding Collective, Wellcome Sanger Institute Tree of Life Management, Samples and Laboratory team, Wellcome Sanger Institute Scientific Operations: Sequencing Operations, Wellcome Sanger Institute Tree of Life Core Informatics team, Tree of Life Core Informatics collective, Darwin Tree of Life Consortium

**Affiliations:** 1 University of Oxford, Oxford, Oxfordshire, England, UK

**Keywords:** Berytinus montivagus, stilt bug, genome sequence, chromosomal, Hemiptera

## Abstract

We present a genome assembly from an individual male *Berytinus montivagus* (stilt bug; Arthropoda; Insecta; Hemiptera; Berytidae). The assembly contains two haplotypes with total lengths of 1,307.32 megabases and 1,100.24 megabases. Most of the haplotype 1 assembly (92.71%) was scaffolded into 17 chromosomal pseudomolecules, including the X and Y sex chromosomes. Haplotype 2 was assembled to scaffold level. The mitochondrial genome has also been assembled, with a length of 15.82 kilobases. This assembly was generated as part of the Darwin Tree of Life project, which produces genomes for eukaryotic species found in Britain and Ireland.

## Species taxonomy

Eukaryota; Opisthokonta; Metazoa; Eumetazoa; Bilateria; Protostomia; Ecdysozoa; Panarthropoda; Arthropoda; Mandibulata; Pancrustacea; Altocrustacea; Allotriocarida; Hexapoda; Insecta; Dicondylia; Pterygota; Neoptera; Eumetabola; Paraneoptera; Hemiptera; Prosorrhyncha; Heteroptera; Euheteroptera; Neoheteroptera; Panheteroptera; Pentatomomorpha; Lygaeoidea; Berytidae; *Berytinus*; *Berytinus montivagus* (Meyer-Dur, 1841) (NCBI:txid1545287)

## Background

*Berytinus* Kirkaldy, 1900 is a genus of stilt bugs in the family Berytidae. Members of the genus are slender bugs with long legs and antennae, and the genus is basically Palaearctic in distribution.

*Berytinus montivagus* (Meyer-Dür, 1841) is a slender stilt bug, approximately 5–7 mm long. The club of the first antennal segment is brown rather than black and the wing membrane is broadly rounded and usually marked with dark brown or black ( Bantock & Botting, 2019; Southwood & Leston, 1959). Southwood and Leston describe it as a central and southern European species ( Southwood & Leston, 1959).

In Britain, *B. montivagus* occurs locally in dry, open habitats and is often associated with medicks on calcareous soils ( Bantock & Botting, 2019). Southwood and Leston recorded it particularly from dry sandy or cindery waste ground where black medick ( *Medicago lupulina*) grows ( Southwood & Leston, 1959). Adults overwinter and mate in spring, with mating and egg laying generally beginning slightly earlier than in *B. crassipes*. The new generation reaches adulthood from July or August onwards ( Bantock & Botting, 2019; Southwood & Leston, 1959).

We present a chromosome-level genome sequence for *Berytinus montivagus*, based on a specimen collected from Wytham Woods, Oxfordshire, UK (Figure 1). This assembly was generated as part of the Darwin Tree of Life Project, which aims to generate high-quality reference genomes for all named eukaryotic species in Britain and Ireland to support research, conservation, and the sustainable use of biodiversity ( Darwin Tree of Life Project Consortium, 2022).

**Figure 1.**
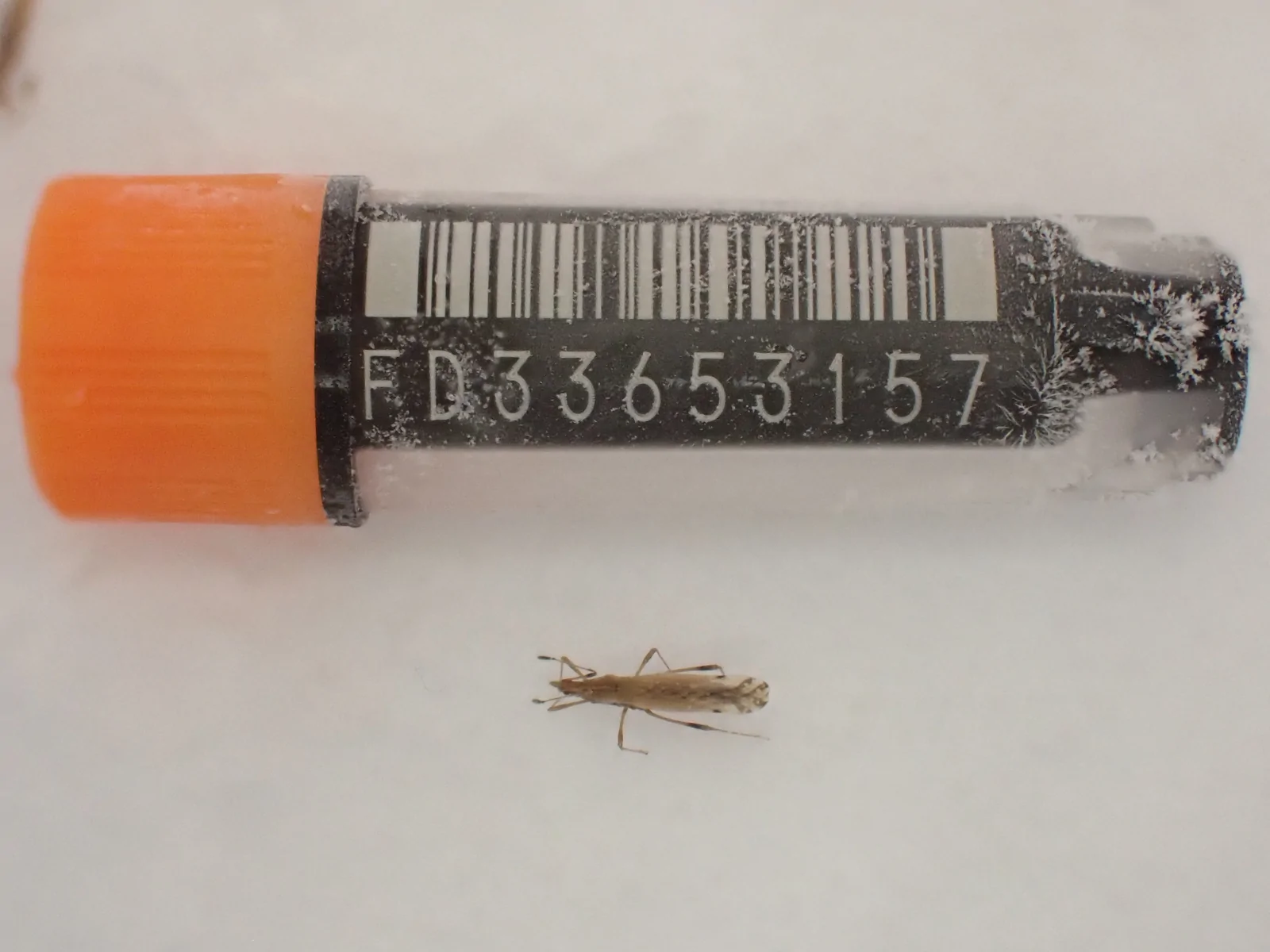
The *B. montivagus* specimen (Ox003405, ihBerMont1) used for genome sequencing.

## Methods

### Sample acquisition and DNA barcoding

The specimen used for genome sequencing was an adult male *Berytinus montivagus* (specimen ID Ox003405, ToLID ihBerMont1; Figure 1). The same specimen was also used for Hi-C sequencing. It was collected from Wytham Woods, Oxfordshire, UK (latitude 51.77, longitude −1.331) on 2023-07-06 by Liam Crowley, who also identified it.

The initial identification was verified by an additional DNA barcoding process according to the framework developed by Twyford *et al.*. (2024). A small sample was dissected from the specimen and stored in ethanol, while the remaining parts were shipped on dry ice to the Wellcome Sanger Institute (WSI) (see the protocol). The tissue was lysed, the COI marker region was amplified by PCR, and amplicons were sequenced and compared to the BOLD database, confirming the species identification ( Crowley *et al.* , 2023). Following whole-genome sequencing, the relevant DNA barcode region was also used alongside the initial barcoding data for sample tracking at the WSI ( Twyford *et al.* , 2024). The standard operating procedures for Darwin Tree of Life barcoding are available on protocols.io.

### Nucleic acid extraction

High molecular weight (HMW) DNA extraction protocols developed by the Tree of Life Core Laboratory at the Wellcome Sanger Institute (WSI) are available on protocols.io ( Howard *et al.*, 2025). Tissue from the ihBerMont1 sample was weighed and triaged to select the appropriate extraction workflow. Tissue from the whole organism was homogenised by PowerMash using a PowerMasher II tissue disruptor. HMW DNA was extracted in the WSI Scientific Operations core using the Automated MagAttract v2 protocol. DNA was sheared into an average fragment size of 12–20 kb following the Megaruptor®3 for LI PacBio protocol. Sheared DNA was purified by automated SPRI (solid-phase reversible immobilisation).

The concentration of the sheared and purified DNA was assessed with a NanoDrop spectrophotometer and a Qubit Fluorometer using the Qubit dsDNA High Sensitivity Assay kit. Fragment size distribution was evaluated by running the sample on the Femto Pulse system. For this sample, final post-shearing DNA measurements included a Qubit concentration of 6.84 ng/μL, an average fragment size of 16.4 kb and a DNA yield of 321.48 ng. The Genomic Quality Number (GQN) was 7.7.

### PacBio HiFi library preparation and sequencing

Library preparation and sequencing took place in the WSI Scientific Operations core. Library DTOL15234391 was prepared with the SMRTbell Prep Kit 3.0 (Pacific Biosciences) according to the manufacturer’s instructions. The kit includes reagents for end repair/A-tailing, adapter ligation, post-ligation SMRTbell bead clean-up, and nuclease treatment. Size selection and clean-up were performed using diluted AMPure PB beads (Pacific Biosciences). DNA concentration was quantified with a Qubit Fluorometer v4.0 (Thermo Fisher Scientific) and the Qubit 1X dsDNA HS assay kit. Final library fragment size was assessed with the Agilent Femto Pulse Automated Pulsed Field CE Instrument (Agilent Technologies) using the gDNA 55 kb BAC analysis kit.

The library was sequenced on a Revio instrument (Pacific Biosciences, California, USA). Prepared libraries with a molarity greater than 2 nM were normalised to 2 nM, and 15 μL was used to make complexes. For libraries with a molarity below 2 nM, the available 10 μL library volume was used without normalisation. Primers were annealed and polymerases were bound to generate circularised complexes following the manufacturer’s instructions. Complexes were purified using 1.2× SMRTbell beads, diluted to a loading concentration of 200–300 pM, and spiked with a Revio sequencing internal control. Sequencing used Revio 25M SMRT cells. SMRT Link software was used to configure and monitor the run and to perform primary and secondary data analysis.

### Hi-C

***Sample preparation and crosslinking***

Hi-C data were generated from the ihBerMont1 whole organism sample using the Arima-HiC v2 kit (Arima Genomics). Following the manufacturer’s instructions, tissue was fixed and DNA crosslinked using TC buffer to a final formaldehyde concentration of 2%. The tissue was homogenised using the Diagnocine Power Masher-II. Crosslinked DNA was digested with a restriction enzyme master mix, biotinylated, and ligated. Clean-up was performed with SPRISelect beads before library preparation. DNA concentration was measured with the Qubit Fluorometer (Thermo Fisher Scientific) and Qubit HS Assay Kit. The biotinylation percentage was estimated using the Arima-HiC v2 QC beads.

***Hi-C library preparation and sequencing***

Biotinylated DNA constructs were fragmented using a Covaris E220 sonicator and size-selected to 400–600 bp using SPRISelect beads. DNA was enriched with Arima-HiC v2 kit Enrichment beads. End repair, A-tailing, and adapter ligation were carried out with the NEBNext Ultra II DNA Library Prep Kit (New England Biolabs), following a modified protocol where library preparation occurs while DNA remains bound to the Enrichment beads. Libraries were amplified with KAPA HiFi HotStart mix and a custom Unique Dual Index (UDI) barcode set (Integrated DNA Technologies). Depending on sample concentration and biotinylation percentage determined at the crosslinking stage, libraries were amplified with 10–16 PCR cycles. Post-PCR clean-up was performed with SPRISelect beads. Libraries were quantified using the AccuClear Ultra High Sensitivity dsDNA Standards Assay Kit (Biotium) and a FLUOstar Omega plate reader (BMG Labtech).

Prior to sequencing, libraries were normalised to 10 ng/μL. Normalised libraries were quantified again to create equimolar and/or weighted 2.8 nM pools. Pool concentrations were checked using the Agilent 4200 TapeStation (Agilent) with High Sensitivity D500 reagents before sequencing. Libraries were sequenced on the , generating paired-end 150-bp reads.

### Genome assembly

Prior to assembly of the PacBio HiFi reads, a database of *k*-mer counts ( *k* = 31) was generated from the filtered reads using FastK. The *k*-mer frequency distribution was analysed with GenomeScope (version 2.1.0) ( Ranallo-Benavidez *et al.*, 2020), providing fitted point estimates of haploid genome size, heterozygosity and repeat content.

The HiFi reads were assembled using Hifiasm in Hi-C phasing mode ( Cheng *et al.*, 2021, 2022), producing two haplotypes. Hi-C reads ( Rao *et al.* , 2014) were mapped to contigs using bwa-mem2 ( Vasimuddin *et al.*, 2019). Contigs were further scaffolded with Hi-C data in YaHS ( Zhou *et al.*, 2023), using the --break option for handling potential misassemblies. The scaffolded assemblies were evaluated using Gfastats ( Formenti *et al.* , 2022), BUSCO ( Manni *et al.*, 2021) and MerquryFK ( Rhie *et al.*, 2020).

The mitochondrial genome was assembled using MitoHiFi ( Uliano-Silva *et al.* , 2023). The MitoHiFi reference was the mitochondrial genome of *Metatropis longirostris* (NC_037373.1).

### Assembly curation

The assembly was decontaminated using the Assembly Screen for Cobionts and Contaminants ( ASCC) pipeline. The flat files and maps for curation were generated with TreeVal. Manual curation was conducted primarily in PretextView and HiGlass ( Kerpedjiev *et al.* , 2018). Scaffolds were visually inspected and corrected as described by Howe *et al*. (2021). The two haplotype assemblies were combined for curation. Manual corrections included 82 breaks and 205 joins. This reduced the scaffold count by 9.3% and increased the scaffold N50 by 4.0%. The curation process is described at sanger-tol/curation-resources. A Hi-C contact map of the haplotype 1 assembly was generated with PretextSnapshot.

### Assembly quality assessment

The MerquryFK tool ( Rhie *et al.*, 2020) was run in a Singularity container ( Kurtzer *et al.*, 2017) to evaluate *k*-mer completeness and assembly quality for both haplotypes using the *k*-mer database ( *k* = 31) computed prior to genome assembly. The analysis outputs included assembly QV scores and completeness statistics.

The haplotype 1 assembly was assessed using BlobToolKit ( Challis *et al.*, 2020). PacBio reads were mapped to the assembly to derive sequence coverage, and BUSCO ( Manni *et al.*, 2021) was used to assess gene-set completeness. DIAMOND BLASTp searches of extracted BUSCO proteins and DIAMOND BLASTx searches of assembly sequences against UniProt Reference Proteomes ( Buchfink *et al.*, 2021), followed by BLASTn searches of sequences without DIAMOND BLASTx hits against the NCBI nt database ( Altschul *et al.*, 1990), supported taxonomic assignment. Coverage, sequence composition, taxonomic assignment and BUSCO results were integrated into a BlobDir for interactive inspection and visualisation. The snail and blob plots were rendered locally from this hosted BlobDir using BlobTk v0.8.3. The scaffold-length radial scale function for the snail plot was set to linear ( Challis & Blaxter, 2026). The blob plot used circular markers with area proportional to scaffold length ( Challis *et al.*, 2020).

## Genome sequence report

### Sequence data

PacBio sequencing of the *B. montivagus* specimen generated 35.35 Gb (gigabases) from 3.74 million reads. These reads were used to assemble the genome. GenomeScope analysis using a *p* = 2 model gave a *k*-mer-based haploid genome size estimate of 1,271.32 Mb, with heterozygosity of 2.59% and repeat content of 63.97%; the full-model fit was 97.72% (Figure 2). The GenomeScope estimates guided expectations for the assembly. The NCBI assembly record reports 27× genome coverage. Hi-C sequencing produced 1,176.10 Gb from 3,894.37 million read pairs, which were used to scaffold the haplotype 1 assembly. Table 1 summarises the specimen and sequencing details.

**Figure 2.**
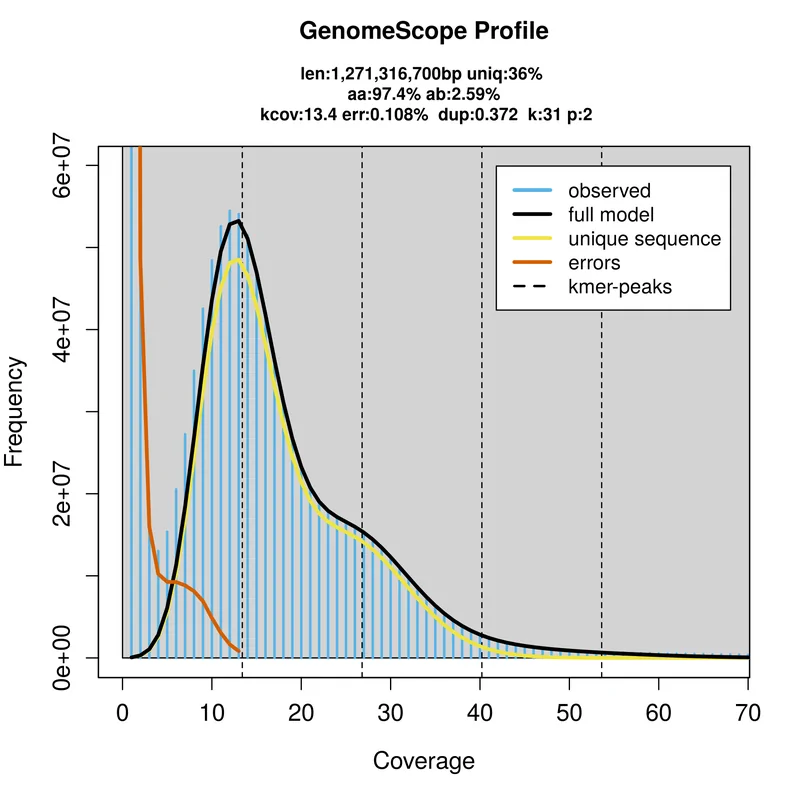
GenomeScope model fitted to the *k*-mer frequency distribution. The plot shows the observed and modelled *k*-mer spectra used to estimate haploid genome size, heterozygosity and repeat content from unassembled sequencing reads.

**Table 1.** Specimen and sequencing data for *B. montivagus* (BioProject PRJEB89328)

**Platform**	**PacBio HiFi**	**Hi-C**
**ToLID**	ihBerMont1	ihBerMont1
**Specimen ID**	Ox003405	Ox003405
**BioSample (source individual)**	SAMEA114644481	SAMEA114644481
**BioSample (tissue)**	SAMEA114645091	SAMEA114645091
**Tissue**	whole organism	whole organism
**Instrument**	Revio	NovaSeqX
**Library ID & run accession(s)**	DTOL15234391: ERR14955683	SQPP-72411-M:G6: ERR14988475
**Read count total**	3.74 million reads	3,894.37 million read pairs
**Base count total**	35.35 Gb	1,176.10 Gb

### Assembly statistics

The genome was assembled into two haplotypes using Hi-C phasing. The haplotype 1 assembly was curated to chromosome level, while haplotype 2 was assembled to scaffold level. The haplotype 1 assembly, which is proposed as the reference assembly for this species, has a total length of 1,307.32 Mb in 630 scaffolds (excluding organelle sequences), with 1,263 gaps, and its scaffold N50 is 72.62 Mb (Table 2). The scaffold auN for the haplotype 1 assembly is 65.6 Mb.

**Table 2.** Genome assembly statistics for *B. montivagus*

**Genome assembly**	**Haplotype 1**	**Haplotype 2**
**Assembly name**	ihBerMont1.hap1.1	ihBerMont1.hap2.1
**Assembly accession**	GCA_965615965.1	GCA_965615715.1
**Assembly level**	chromosome	scaffold
**Span (Mb)**	1,307.32	1,100.24
**Number of chromosomes**	17	-
**Number of contigs**	1,893	1,706
**Contig N50 (Mb)**	1.34	1.51
**Number of scaffolds (excluding organelle sequences)**	630	606
**Scaffold N50 (Mb)**	72.62	70.8
**Scaffold auN (Mb)**	65.6	65.3
**Longest scaffold length (Mb)**	103.8	102.49
**Sex chromosomes**	X and Y	-
**Organelles**	Mitochondrial genome: 15.82 kb	-

Most of the haplotype 1 assembly sequence (92.71%) was assigned to 17 chromosome-level scaffolds, representing 15 autosomes and the X and Y sex chromosomes. These chromosome-level scaffolds, confirmed by Hi-C data, are named according to size (Figure 3; Table 3).

The mitochondrial genome was also assembled (length 15.82 kb, OZ278054.1). This sequence is included as a contig in the multifasta file of the haplotype 1 genome submission and as a standalone record.

**Figure 3.**
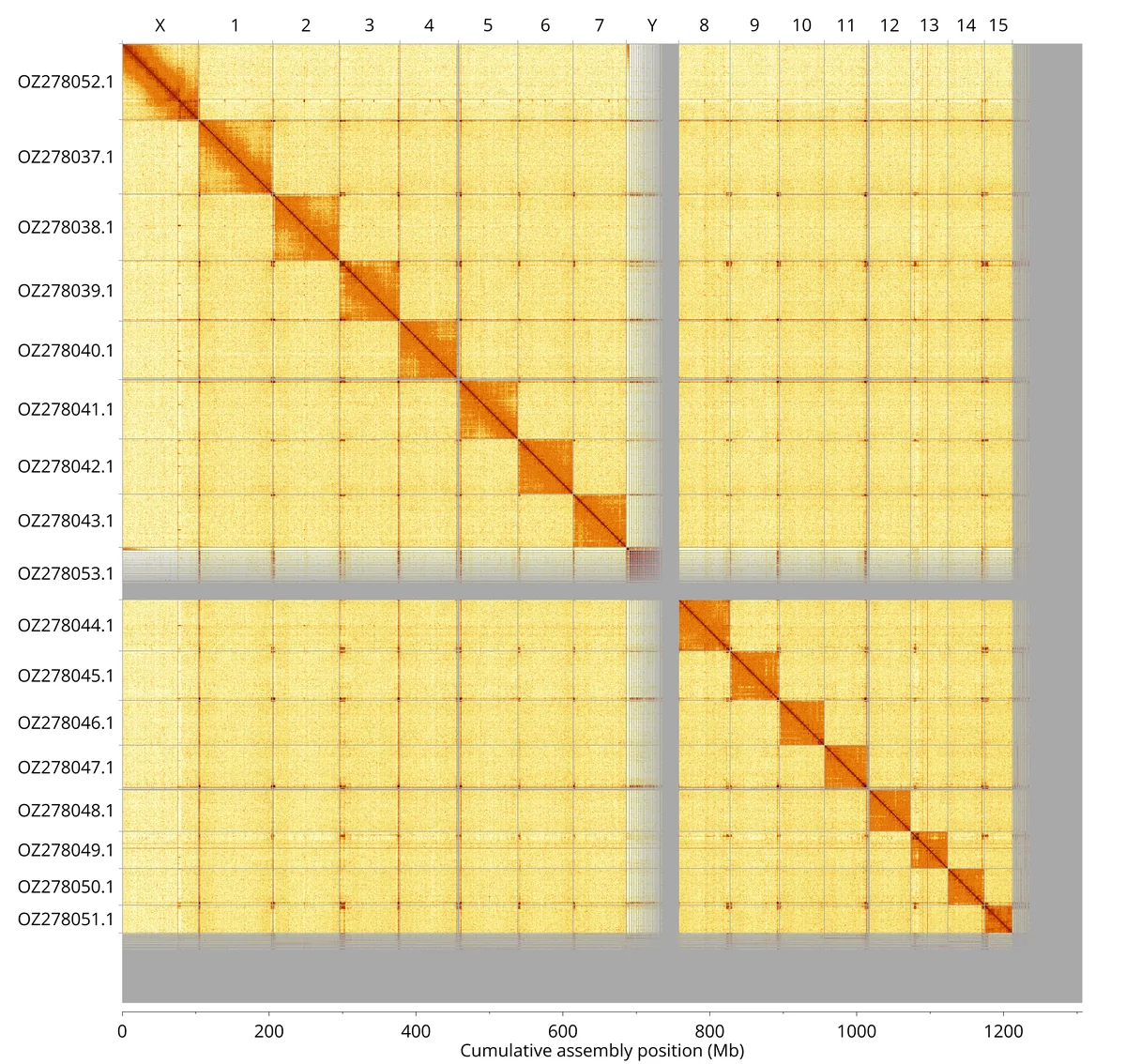
Hi-C contact map of the *B. montivagus* haplotype 1 assembly. Assembled chromosomes are shown in order of size and labelled along the axes, with a megabase scale shown below. The plot was generated using PretextSnapshot.

**Table 3.** Chromosomal pseudomolecules in the haplotype 1 genome assembly of *B. montivagus* ihBerMont1

**INSDC accession**	**Molecule**	**Length (Mb)**	**GC%**
OZ278037.1	1	101.13	38.50
OZ278038.1	2	90.52	38.50
OZ278039.1	3	82.55	38.50
OZ278040.1	4	80.73	38.50
OZ278041.1	5	80.14	38.50
OZ278042.1	6	74.84	38.50
OZ278043.1	7	72.62	38.50
OZ278044.1	8	70.15	38.50
OZ278045.1	9	66.92	38.50
OZ278046.1	10	61.15	38.50
OZ278047.1	11	61.10	38.50
OZ278048.1	12	56.51	38.50
OZ278049.1	13	50.56	39
OZ278050.1	14	50	38.50
OZ278051.1	15	38	38.50
OZ278052.1	X	103.80	38.50
OZ278053.1	Y	71.34	49

### Assembly quality metrics

For the haplotype 1 assembly, the estimated QV is 60.6, and for the haplotype 2 assembly, 60.5. When the two haplotypes are combined, the assembly achieves an estimated QV of 60.5. The *k*-mer completeness is 68.76% for the haplotype 1 assembly, 61.33% for the haplotype 2 assembly, and 97.74% for the combined haplotypes (Figure 4).

**Figure 4.**
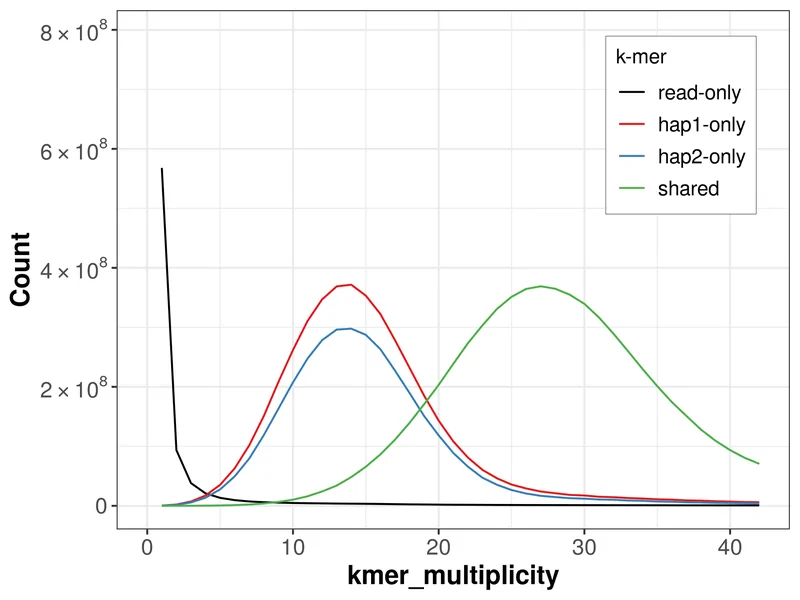
Evaluation of *k*-mer completeness using MerquryFK. This plot illustrates the recovery of *k*‐mers from the original read data in the haplotype 1 assembly, the haplotype 2 assembly and the combined haplotypes. The horizontal axis represents *k*‐mer multiplicity, and the vertical axis shows the number of *k*‐mers. The black curve represents *k*‐mers that appear in the reads but are not assembled. The green curve corresponds to *k*‐mers shared by both haplotypes, and the red and blue curves show *k*‐mers found only in one of the haplotypes.

BUSCO analysis using the hemiptera_odb10 reference set ( *n* = 2,510) identified 98.4% of the expected gene set (single = 97.0%, duplicated = 1.4%) in the haplotype 1 assembly. For the haplotype 2 assembly, BUSCO v.5.8.3 analysis identified 89.8% of the expected gene set (single = 88.9%, duplicated = 0.8%).

The snail plot in Figure 5 provides a summary of assembly contiguity, cumulative scaffold count, sequence composition and BUSCO completeness for the haplotype 1 assembly ( Challis & Blaxter, 2026). The relative sizes of the longest scaffold, scaffold N50 and N90, together with the dark-grey scaffold-length distribution, show how much of the assembly is contained in long scaffolds. The outer composition track shows variation in GC, AT and N content, while the central track shows the cumulative scaffold count and the BUSCO inset summarises gene-set completeness.

The blob plot in Figure 6 displays scaffolds from the haplotype 1 assembly by GC proportion and base coverage. Marker area is proportional to scaffold length, and colour indicates taxonomic assignment ( Challis *et al.*, 2020).

**Figure 5.**
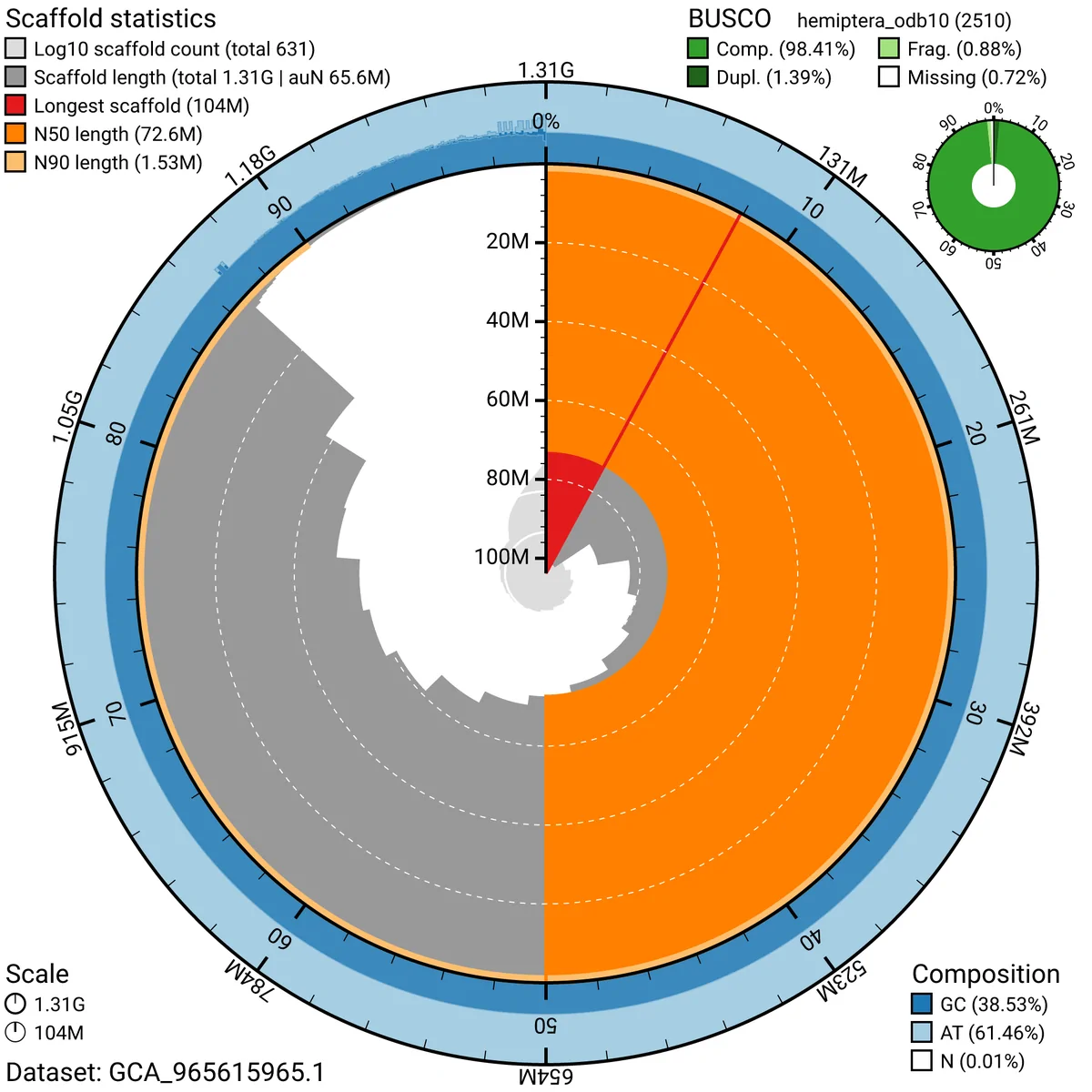
Assembly metrics for ihBerMont1.hap1.1, the haplotype 1 assembly. The BlobToolKit snail plot provides an overview of assembly metrics and BUSCO gene completeness. The circumference represents the length of the haplotype 1 assembly, and the main plot is divided into 1,000 bins around the circumference. The outermost blue tracks display the distribution of GC, AT, and N percentages across the bins. Scaffolds are arranged clockwise from longest to shortest and are depicted in dark grey. Scaffold length is shown on a linear radial scale, scaled to the longest scaffold. Red shading indicates the longest scaffold, and the deeper orange and pale orange shading represent the N50 and N90 lengths. The summary statistics include scaffold auN, the area under the scaffold Nx curve. The light grey spiral at the centre shows the cumulative scaffold count on a logarithmic scale. A summary of complete, fragmented, duplicated, and missing BUSCO genes in the hemiptera_odb10 set is presented at the top right. The assembly can be explored interactively in the BlobToolKit viewer.

**Figure 6.**
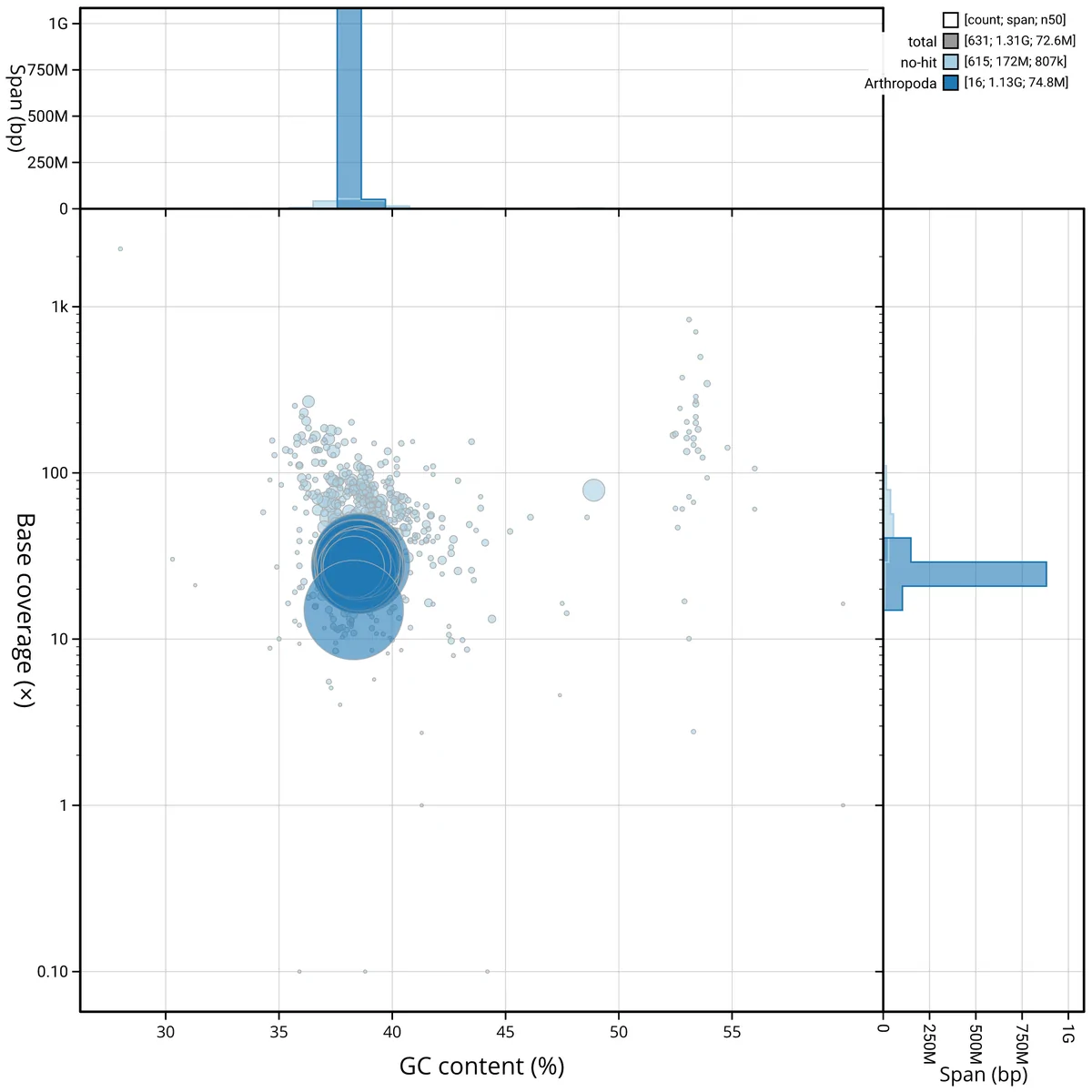
BlobToolKit blob plot for ihBerMont1.hap1.1, the haplotype 1 assembly. The plot shows base coverage (vertical axis) and GC content (horizontal axis). Circles represent scaffolds; marker area is proportional to scaffold length, and colour indicates taxonomic assignment. The histograms along the axes display the total length of sequences distributed across different levels of coverage and GC content. The assembly can be explored interactively in the BlobToolKit viewer.

Table 4 lists the assembly metric benchmarks adapted from Rhie *et al.*. (2021) and the Earth BioGenome Project (EBP) Report on Assembly Standards. The three-part EBP metric, calculated for the haplotype 1 assembly, is **6.C.Q60**.

**Table 4.** Earth BioGenome Project summary metrics for the *B. montivagus* assembly

**Measure**	**Value**	***Benchmark***
EBP summary (haplotype 1)	6.C.Q60	*6.C.Q40*
Contig N50 length	1.34 Mb	*> 1 Mb*
Scaffold N50 length	72.62 Mb	*= chromosome N50*
Consensus quality (QV)	Haplotype 1: 60.6; haplotype 2: 60.5; combined: 60.5	*≥ 40*
*k*-mer completeness	Haplotype 1: 68.76%; Haplotype 2: 61.33%; combined: 97.74%	*> 90% (combined assembly)*
BUSCO	Haplotype 1: C:98.4% [S:97.0%, D:1.4%], F:0.9%, M:0.7%, n:2,510; Haplotype 2: C:89.8% [S:88.9%, D:0.8%], F:0.9%, M:9.3%, n:2,510	*S > 90%; D < 5%*
Percentage of assembly assigned to chromosomes	92.71%	*≥ 90%*

**Notes:** The EBP summary uses log10(Contig N50); chromosome-level (C) or log10(Scaffold N50); Q (Merqury QV). For *k*-mer completeness, the benchmark is assessed using the combined assembly value. BUSCO v5.8.3 using the hemiptera_odb10 lineage dataset: C=complete; S=single-copy; D=duplicated; F=fragmented; M=missing; n=orthologues.

### Data availability

European Nucleotide Archive: Berytinus montivagus. Accession number PRJEB89328; https://identifiers.org/ena.embl/PRJEB89328. The genome sequence is released openly for reuse. The *B. montivagus* genome sequencing initiative is part of the Darwin Tree of Life Project ( PRJEB40665) and the Sanger Institute Tree of Life Programme ( PRJEB43745). All raw sequence data and the haplotype assemblies have been deposited in INSDC databases. The genome will be annotated using available RNA-Seq data and presented through the Ensembl pipeline at the European Bioinformatics Institute. Raw data and assembly accession identifiers are reported in Table 1 and Table 2.

Production code used in genome assembly at the WSI Tree of Life is available at https://github.com/sanger-tol. Table 5 lists software versions used in this study.

**Table 5.** Software versions and sources

**Software**	**Version**	**Source**
BLAST	2.14.0	https://ftp.ncbi.nlm.nih.gov/blast/executables/blast+/
BlobToolKit	4.4.6	https://github.com/genomehubs/blobtoolkit
BlobTk (hosted BlobDir)	0.5.1	https://github.com/genomehubs/blobtk
BlobTk (snail and blob plot renderer)	0.8.3	https://github.com/genomehubs/blobtk
BUSCO	5.8.3; 6.1.0	https://gitlab.com/ezlab/busco
bwa-mem2	2.2.1	https://github.com/bwa-mem2/bwa-mem2
DIAMOND	2.1.8	https://github.com/bbuchfink/diamond
fasta_windows	0.2.4	https://github.com/tolkit/fasta_windows
FastK	1.2	https://github.com/thegenemyers/FASTK
GenomeScope	2.1.0	https://github.com/tbenavi1/genomescope2.0
Gfastats	1.3.6	https://github.com/vgl-hub/gfastats
Hifiasm	0.19.8-r603	https://github.com/chhylp123/hifiasm
HiGlass	1.13.4	https://github.com/higlass/higlass
MerquryFK	1.1.0-c1	https://github.com/thegenemyers/MERQURY.FK
Minimap2	2.28-r1209	https://github.com/lh3/minimap2
MitoHiFi	3.2.2	https://github.com/marcelauliano/MitoHiFi
MultiQC	1.14; 1.17 and 1.18	https://github.com/MultiQC/MultiQC
Nextflow	24.10.4	https://github.com/nextflow-io/nextflow
PretextSnapshot	0.0.6	https://github.com/sanger-tol/PretextSnapshot
PretextView	1.0.3	https://github.com/sanger-tol/PretextView
samtools	1.21	https://github.com/samtools/samtools
sanger-tol/ascc	0.1.0	https://github.com/sanger-tol/ascc
sanger-tol/blobtoolkit	v0.8.0	https://github.com/sanger-tol/blobtoolkit
sanger-tol/curationpretext	1.4.2	https://github.com/sanger-tol/curationpretext
Seqtk	1.4-r122	https://github.com/lh3/seqtk
Singularity	3.9.0	https://github.com/sylabs/singularity
TreeVal	1.3.0	https://github.com/sanger-tol/treeval
YaHS	1.2.2	https://github.com/c-zhou/yahs

### Author information

Contributors are listed at the following links:

- Members of the University of Oxford and Wytham Woods Genome Acquisition Lab
- Members of the Darwin Tree of Life Barcoding collective
- Members of the Wellcome Sanger Institute Tree of Life Management, Samples and Laboratory team
- Members of Wellcome Sanger Institute Scientific Operations – Sequencing Operations
- Members of the Wellcome Sanger Institute Tree of Life Core Informatics team
- Members of the Tree of Life Core Informatics collective
- Members of the Darwin Tree of Life Consortium

### Wellcome Sanger Institute – Legal and Governance

The materials that have contributed to this genome note have been supplied by a Darwin Tree of Life Partner. The submission of materials by a Darwin Tree of Life Partner is subject to the **‘Darwin Tree of Life Project Sampling Code of Practice’**, which can be found in full on the Darwin Tree of Life website. By agreeing with and signing up to the Sampling Code of Practice, the Darwin Tree of Life Partner agrees they will meet the legal and ethical requirements and standards set out within this document in respect of all samples acquired for, and supplied to, the Darwin Tree of Life Project. Further, the Wellcome Sanger Institute employs a process whereby due diligence is carried out proportionate to the nature of the materials themselves, and the circumstances under which they have been/are to be collected and provided for use. The purpose of this is to address and mitigate any potential legal and/or ethical implications of receipt and use of the materials as part of the research project, and to ensure that in doing so we align with best practice wherever possible. The overarching areas of consideration are:

- Ethical review of provenance and sourcing of the material
- Legality of collection, transfer and use (national and international)

Each transfer of samples is further undertaken according to a Research Collaboration Agreement or Material Transfer Agreement entered into by the Darwin Tree of Life Partner, Genome Research Limited (operating as the Wellcome Sanger Institute), and in some circumstances, other Darwin Tree of Life collaborators.
