# Supplementary material for: The genome sequence of the stilt bug, Berytinus montivagus (Meyer-Dur, 1841) (Hemiptera: Berytidae)
Source: Wellcome Open Res. 2026 Oct 2;11:788. [Version 1] doi: 10.12688/wellcomeopenres.28016.1 (PMC13687739; doi:10.12688/wellcomeopenres.28016.1)
Supplement: Peer review history

# Reviewer report: Reviewer response for version 1

**DOI:** 10.21956/wellcomeopenres.30821.r172377

**Published:** 2026-10-21

**Authors:** Gabriela Jijon

**Affiliations:** 1 Brigham Young University, Provo, Utah, USA

This Genome Note reports the high-quality genomic assembly of *Berytinus montivagus* and includes two haplotypes, one assembled into chromosomal pseudomolecules and the other to scaffold level. This reference genome was generated to support the Darwin Tree of Life Project's aim of sequencing all eukaryotic species in Britain and Ireland. The publication provides background information on the morphology and ecology of *B. montivagus*. The background provided on the species is succinct and informative. However, the rationale and importance of sequencing this stilt bug could have been expanded to highlight why sequencing this taxon is particularly relevant. 

 The Methods section could have been enhanced by including additional details. For example, indicating the recorded weight during sample triage and including further details on the prep and sequencing would have strengthened this section. Explicitly stating whether the HiFi library molarity was above or below 2 nM, how many Revio 25M SMRT cells were used, and how many HiC libraries were produced and sequenced would also have enhanced this section. The following sentence needs revision as it omits the sequencing system used: “Libraries were sequenced on the , generating paired-end 150-bp reads.” Other details that could have been included, such as the ASCC pipeline version used, the aligner/mapper (e.g., minimap2) used to produce the input file for Blobtools, and whether contaminant sequences were filtered, would have strengthened this section. Using the term “whole organism sample” when it includes a partial sample of the whole organism is a bit confusing, and I would recommend rephrasing this as “partial sample of the whole organism” to improve clarity. 

 In the Genome Sequence Report section, the link in Table 1 to the HiC library ID and run accession page (SQPP-72411-M:G6: ERR14988475) reported no records at the time of this review. The tables and figures included were relevant and well presented. 

 This publication expands our genomic resources for insects and can help us better understand their evolution and diversity. I enjoyed reviewing this Genome note!

Are the datasets clearly presented in a usable and accessible format, and the assembly and annotation available in an appropriate subject-specific repository?

Yes

Are sufficient details of the sequencing and extraction, software used, and materials provided to allow replication by others?

Partly

Are the rationale for sequencing the genome and the species significance clearly described?

Yes

Are the protocols appropriate and is the work technically sound?

Yes

Reviewer Expertise:

My area of research is insect genomics and transcriptomics, particularly focusing on caddisflies.

I confirm that I have read this submission and believe that I have an appropriate level of expertise to confirm that it is of an acceptable scientific standard.

# Reviewer report: Reviewer response for version 1

**DOI:** 10.21956/wellcomeopenres.30821.r172382

**Published:** 2026-10-16

**Authors:** Stuart J.E. Baird

**Affiliations:** 1 Academy of Sciences of the Czech Republic, Národní, Czech Republic

The genome report follows the excellent template of DToL. I find two small issues:

 Section “Hi-C library preparation and sequencing” final line:

 “Libraries were sequenced on the , generating paired-end 150-bp reads.”

 [Sequencing machine name omitted]

 Section “Genome assembly” final line:

  “The mitochondrial genome was assembled using MitoHiFi (Uliano-Silva et al., 2023). The MitoHiFi reference was the mitochondrial genome of Metatropis longirostris (NC_037373.1).”

 [It is unclear whether the mt genome is assembled de novo, or using a reference, because a reference is mentioned without telling the reader what it is used for…clarify]

Are the datasets clearly presented in a usable and accessible format, and the assembly and annotation available in an appropriate subject-specific repository?

Yes

Are sufficient details of the sequencing and extraction, software used, and materials provided to allow replication by others?

Yes

Are the rationale for sequencing the genome and the species significance clearly described?

Yes

Are the protocols appropriate and is the work technically sound?

Yes

Reviewer Expertise:

Admixture genomics

I confirm that I have read this submission and believe that I have an appropriate level of expertise to confirm that it is of an acceptable scientific standard, however I have significant reservations, as outlined above.

# Reviewer report: Reviewer response for version 1

**Published:** 2026-10-02

**Authors:** Automated Benchmarking

**Affiliations:** 1 Wellcome Sanger Institute, Hinxton, UK

As this article is a Genome Note, Automated Benchmarking has been used to determine whether the reported assembly passes four defined checks established by the Earth BioGenome Project: the raw and assembled data are publicly available, the Earth BioGenome Project three-part metric is met, the k-mer completeness exceeds 90%, and the BUSCO score passes the stated threshold. For Berytinus montivagus (ihBerMont1), all four checks passed. Data available: pass. ENA study PRJEB89328, raw-read accession ERR14955683, and assembly accessions GCA_965615965.1 and GCA_965615715.1 are public. Earth BioGenome Project three-part metric: pass. Haplotype 1 is reported as 6.C.Q60, meeting the 6.C.Q40 reference standard. k-mer completeness: pass. The combined haplotype assemblies have 97.74% k-mer completeness, exceeding the Earth BioGenome Project target of 90%. BUSCO: pass. Haplotype 1, which is proposed as the reference assembly for this species, has 98.4% complete BUSCOs (97.0% single-copy and 1.4% duplicated). BUSCO software version: 5.8.3; lineage dataset: hemiptera_odb10. It meets the target of greater than 90% complete and single-copy BUSCOs. This decision uses values reported in the Genome Note and public accession records.

Reviewer Expertise:

NA

I confirm that I have read this submission and believe that I have an appropriate level of expertise to confirm that it is of an acceptable scientific standard.
